# Screw Withdrawal Resistance from WPC Profiles Used in Door Frame Production

**DOI:** 10.3390/ma19071351

**Published:** 2026-03-29

**Authors:** Zbigniew Potok, Zdzisław Kwidziński, Marta Pędzik, Krzysztof Wiaderek, Barbara Prałat, Tomasz Rogoziński

**Affiliations:** 1 Department of Furniture Design, Faculty of Forestry and Wood Technology, Poznań University of Life Sciences, 60-637 Poznań, Poland; zdzislaw_kwidzinski@porta.com.pl (Z.K.); marta.pedzik@pit.lukasiewicz.gov.pl (M.P.); krzysztof.wiaderek@up.poznan.pl (K.W.); barbara.pralat@up.poznan.pl (B.P.); tomasz.rogozinski@up.poznan.pl (T.R.); 2 PORTA KMI Poland, 84-239 Bolszewo, Poland; 3 Łukasiewicz Research Network—Poznań Institute of Technology, 6 Ewarysta Estkowskiego St., 61-755 Poznań, Poland

**Keywords:** screw pull-out resistance, wood-based materials, wooden doors, wood screws

## Abstract

This study investigates the screw withdrawal resistance (SWR) of hollow wood–plastic composite (WPC) door frames, which serve as moisture-resistant alternatives to traditional wood-based materials. The tested WPC, characterised by a density of 1.33 g/cm^3^ and a polymer-bound lignocellulosic filler, exhibits superior dimensional stability and low water absorption—under 4% after 24 h of immersion. The research focuses on how the unique chambered geometry of these industrial profiles affects the anchoring of 20 mm conical wood screws used to mount essential fittings such as hinges and lock catches. The SWR was determined using a universal testing machine in accordance with the modified EN 320 standards. Results indicate that the installation location within the profile significantly dictates load-bearing capacity: the band profile (lock catch) achieved an average SWR of 525.65 N, while the beam profile (hinge) averaged only 275.25 N. This performance gap arises because screws anchor only into internal “ribs” rather than the full material depth. Since these values are considerably lower than those of traditional particleboard (~1364–1775 N), the study highlights a critical need to optimise screw dimensions to ensure the structural stability and safety of hollow WPC door systems.

## 1. Introduction

Growing demands for the durability of building materials are driving an intensive search for solutions that combine water resistance with dimensional stability and appropriate mechanical properties. In building joinery elements, such as some door frames, the material is exposed to periodic moisture, temperature changes and prolonged exposure to air with high relative humidity. These factors can lead to swelling, deformation and degradation of the material, which directly affects the durability of the structure, the aesthetics of the products and their use. Traditional wood-based materials, despite their widespread use in such conditions, may show a gradual loss of performance parameters [1]. For this reason, increasing attention is being paid to composite materials in which the wood phase is partially isolated from the external environment by a polymer matrix, which reduces the negative impact of moisture while maintaining the beneficial properties of wood-based materials.

Wood–plastic composites (WPCs) are a group of materials made from a combination of lignocellulosic filler, usually in the form of wood flour or fine wood particles, with a thermoplastic polymer matrix [2]. The wood raw material used includes both classic softwood and hardwood flour, as well as bark particles and other lignocellulosic waste materials of plant origin, which in many cases are by-products of wood processing [3,4]. Numerous studies indicate that particles with dimensions of approximately 177 to 250 μm are advantageous for processing and mechanical properties, but this depends on the intended use of the WPC [5]. This filler size promotes a relatively homogeneous composite structure and allows the polymer to effectively surround the wood particles during the processing. At the same time, the use of wood waste with controlled granulation allows for the utilisation of material fractions that would be difficult to use in other applications. The combination of lignocellulosic filler, e.g., pine bark [6], wood flour [7] or waste wood and crop straw [8] with polymers, including recycled plastics, enables the simultaneous management of wood and plastic waste and is in line with the concept of rational raw material management and reducing the amount of waste sent to landfill. In this respect, WPCs can be treated as materials that combine functionality with environmental considerations, as they allow waste materials to be reused in products with a relatively long-life cycle [6]. The presence of the polymer phase limits direct contact between wood particles and water, thereby improving dimensional stability and reducing water absorption compared to other materials. However, the mechanical properties of such composites depend largely on the content of wood particles, their size, shape and the quality of interfacial interactions between wood and polymer. As a result, even slight changes in the filler’s structure can translate into noticeable differences in the material’s behaviour under long-term loads. In addition, the amount of filler used affects its processing efficiency, as it has been proven that an increase in wood flour content from 30 to 50% resulted in greater composite strength, but also greater cutting forces [9].

A special aspect that is less frequently discussed in the context of WPC is the possibility of using very fine fractions of wood particles generated during the mechanical processing of wood and wood-based panels. Wood dust is considered harmful to health, and its impact depends largely on particle size and exposure time [10]. In the case of WPC, it is possible to permanently bind very fine lignocellulosic fractions in a polymer matrix, which limits their release into the environment during the use of the finished product. This approach can be considered as a way of managing difficult waste and reducing the emission of fine particles into the environment. At the same time, the presence of very fine filler particles can affect the local structure of the composite, the degree of structure compaction and the stress distribution in load concentration zones. From the point of view of structural elements, such as door frames, the behaviour of the material in the area where mechanical fasteners are embedded may be particularly important.

In applications such as door frames, WPCs compete with traditional materials such as solid wood, particleboard, and MDF (medium-density fibreboard). Despite their widespread use and well-developed production technologies, these materials have limited resistance to prolonged exposure to moisture, which can lead to dimensional changes, reduced strength and deterioration of mechanical joints. In the case of wood-based panels, an additional factor is the presence of adhesives and a porous structure, which promotes water absorption and degradation in conditions of increased humidity. Thanks to the presence of plastics, WPCs are characterised by significantly lower water absorption, typically gain up to 3% mass gain after 24 h immersion compared to approximately 24% for solid wood, as well as greater dimensional stability [11]. This reduced uptake results from the hydrophobic polymer phase, which encapsulates wood particles and limits moisture penetration [5,12]. As a result, WPC panels are also more resistant to fungi, mould, and insects, as the limited availability of moisture inhibits the growth of microorganisms, making them an attractive solution in environments with increased high humidity. Although WPCs may still show some moisture sensitivity compared to neat polymers, they outperform conventional wood-based materials in humid environments, while not reaching the moisture resistance of pure plastics [13]. From the point of view of the functionality of door frames, however, it is not only environmental resistance that is crucial, but also the material’s ability to transfer loads transmitted by mechanical fasteners and to maintain adequate rigidity in the installation areas. The ability to hold screws and other fasteners is one of the basic conditions for the safe and durable installation of door frames. Mechanical connections in this type of element transfer operational loads, ensure the correct positioning of the door leaf and affect the overall stability of the structure.

Screws used in door frames play an important structural role, ensuring the transfer of loads from the weight of the door leaf, hinge operation, and forces generated during door use. Their role is crucial to both the geometric stability of the door system and its resistance to dynamic impacts. According to the results of material tests on wood composites and the structure of mechanical fasteners, the properties of the substrate material into which the screws are inserted determine their load-bearing capacity, stiffness and susceptibility to fatigue degradation [14].

Screws in door frames transfer static loads from the weight of the door leaf and dynamic loads associated with opening and closing cycles. In wood-based materials, including polymer structures reinforced with lignocellulosic fibres, the mechanical anchoring of the fastener depends on the substrate’s physical properties and anisotropy, as well as on the geometric parameters of the screw itself [15]. Properly selected thread, diameter and anchoring depth determine not only the load-bearing capacity, but also the stability of the connection during long-term use [16].

The selection of screws for door frames made of wood, PVC or metal materials requires consideration of both the rheological and strength properties of the base material and environmental factors. These include variability in humidity, the possibility of corrosion, and stresses resulting from the operation of fittings. Screws intended for polymer substrates, such as PVC, must have a geometry that reduces the risk of local cracks and deformation of the construction material. For wood-based substrates, parameters that enable effective anchoring within the fibrous structure are crucial.

In practical carpentry applications, including window and door joinery, the quality of screw connections translates into the functional durability of doors, the stability of hinge settings, adjustability and resistance to structural fatigue. Thanks to their design, screw connections allow easy adjustment of hinges and efficient maintenance and replacement of components without interfering with the entire frame structure. Given the durability of mechanical fasteners, the correct selection of screws is an important factor in the engineering of durable door systems and their effective operation. Although there are studies concerning the screw pull-out resistance of wood and wood-based materials, including analyses of the influence of screw geometry, penetration depth and material structure, studies concerning WPCs are still limited and are most often conducted on homogeneous, laboratory-prepared specimens [17,18,19]. As a result, the available data do not fully reflect the behaviour of industrial WPC products, which are frequently non-uniform in cross-sections and may contain hollow chambers and locally varying material structure.

Despite the growing interest in WPCs in construction, their behaviour in the context of fastener pull-out in actual building joinery elements is relatively rarely analysed in the literature. In this study, fragments of WPC door frames from industrial production were tested. Unlike typical material-oriented studies, the present work focuses on commercially manufactured, non-homogeneous WPC profiles, thereby directly addressing the mechanical efficiency of fastener anchoring in real structural components. This approach enables a more realistic assessment of screw performance under practical application conditions and provides data relevant for engineering design and material selection. This approach allows evaluating the behaviour of the actual product used in practice, considering the technological conditions of mass production and the repeatability of parameters.

The use of a new type of material to produce door frames necessitates testing of the material from which they will be made. The differences in the materials used to produce door frames are visible to the naked eye. Particleboard is a solid material, while WPC is hollow. This makes it necessary to check whether the screws used to mount fittings during the production of particleboard door frames will be able to transfer similar loads when used in the production of WPC profile door frames. Therefore, the objective of this study is to evaluate the screw pull-out resistance in industrial WPC door frame profiles and to verify whether existing fastening solutions ensure adequate load-bearing capacity in comparison with conventional materials. These tests are intended to determine whether the screws used so far are sufficient to ensure the door frame meets the required strength class.

## 2. Materials and Methods

### 2.1. WPC Profiles

The tests were carried out on solid, sanded WPC boards manufactured by Pietrucha International Sp. z o.o. (Błaszki, Poland). The elements were used in their commercial state, without any interference with their material composition. The tested material is a wood–plastic composite with the properties given below by the manufacturer: a density of 1.33 g/cm^3^ and a weight-to-length ratio of approximately 4948 g/m. The material is characterised by limited water absorption. Absorption after 24 h of immersion does not exceed 4%, while the change in thickness determined by swelling after 7 days of immersion in water is no more than 1%. The Brinell hardness in an air-dry state is at least 110 N/mm^2^, and after 7 days of exposure to water, it remains at least 85 N/mm^2^. The flexural strength is not less than 36 MPa, the flexural modulus reaches a value of at least 5000 MPa, and the compressive strength is not less than 8 MPa.

**Figure 1:**
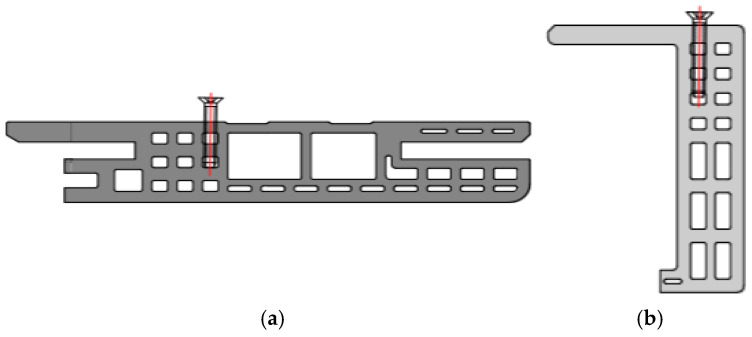
Samples before testing: (**a**) screw location in the beam, (**b**) screw location in the clamp.

Two types of samples were prepared depending on the location of the screws in the finished door frame. The cross-section of the profiles used to produce door frames, together with the location of the screws used to fix the fittings, is shown in Figure 1.

Samples marked with the symbol A show where the screws responsible for fixing the hinges are mounted in the WPC profile beam, while in sample B, these screws are used to fix the lock catch.

### 2.2. Screws

**Figure 2:**
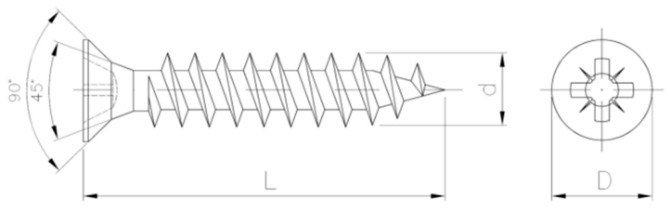
Screw used for SWR measurements.

The experiment used fully threaded conical wood screws with a length of L = 20 mm, a thread diameter of d = 2.5 mm and a head diameter of D = 4.8 mm. The selection of screws was made based on information from the frame producer. The same type of screws is used to produce door frames for fixing fittings. The screws were installed in the same locations in the WPC profile cross-section where fittings are installed during the production of door frames. Figure 2 shows the screw used to prepare the samples.

### 2.3. Samples for SWR Tests

To prepare the samples, one board of each WPC profiles were divided into 5 cm-wide elements. Then, a pilot hole was drilled in the centre of the element across its width, exactly at the point on the profile where the screws for mounting the fittings are installed. Pilot holes were made with a 2 mm diameter drill on a through-feed machining centre that is part of the G. Kraft Maschinenbau GmbH technological line. Eight samples were prepared from each of the two sample variants for SWR testing. In accordance with the production method for door frames, pilot holes were drilled before screws were inserted.

### 2.4. SWR Measurements

**Figure 3:**
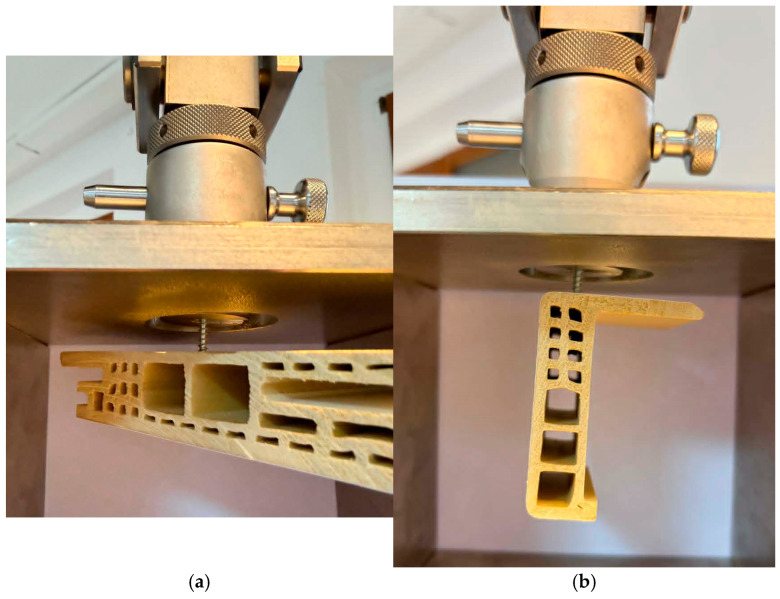
Experimental setup for withdrawal measurements (**a**) beam sample, (**b**) the clamp sample.

The screw pull-out force was determined using a Zwick/Roel Z010 universal testing machine (Zwick/Roel, Ulm, Germany). The tests were carried out in accordance with EN 320 [20] with minor modifications to adapt the method to the prepared samples. The modifications involved using a different type of screw than the standard and modifying the shape of the samples. The tested material was WPC profiles, not WPCs, so individual profiles were not modified, only divided into samples. The measurement procedure itself and the method of removing the screws were carried out in accordance with the guidelines of the standard. The test samples were placed in a screw holder and then stretched at 10 mm/min speed using a preload of 5 N. The samples during the tests are shown in Figure 3.

### 2.5. Statistical Analysis

Results were obtained for each sample, enabling a direct comparison of the influence of surface geometry under the same measurement conditions. Prior to statistical analysis, basic descriptive statistics were calculated, including the arithmetic mean and standard deviation. The significance of differences between surfaces was assessed using two types of statistical analysis. The first one was Student’s *t*-test for dependent samples, assuming a significance level of α = 0.05; the second one was a Shapiro–Wilk test. Both were performed in R studio (ver. 2025.09.0+418).

## 3. Results and Discussion

**Figure 4:**
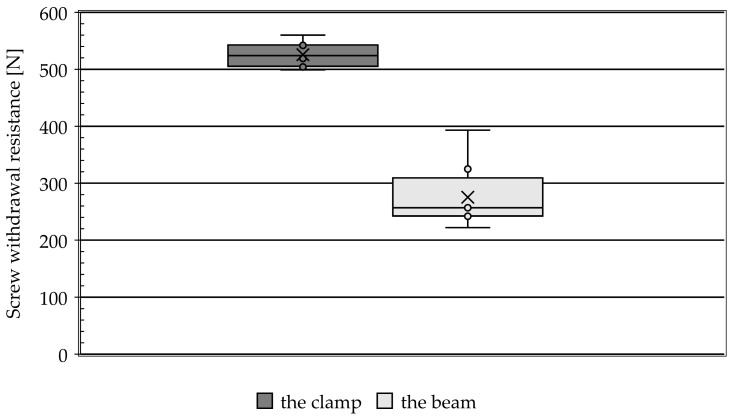
A box plot comparing screw withdrawal resistances in the series.

Figure 4 shows a box plot summarising the distribution of forces obtained during the measurement of the withdrawal force of the screw. The mean in the series is marked with the symbol ‘x’ and the median with a horizontal line. The median and mean indicate the central tendency, and the interquartile range (IQR) represents the spread of the data. The length of the whiskers reflects the distribution’s skewness.

Samples made from the band profile showed higher SWR values, averaging 525.65 N, compared to samples from the beam, which averaged 275.25 N. The difference in medians is even greater, with 524 N for the band and 257 N for the beam.

The statistical analysis revealed clear, statistically significant differences between narrow and wide surfaces. The difference between the means of the analysed surfaces was 250.38, indicating a pronounced geometric effect. Student’s *t*-test confirmed the statistical significance of the observed differences (t(7) = 14.18; *p* = 2.06 × 10^−6^), which means that the probability of such large discrepancies occurring by chance is negligible. The results clearly indicate that changing the surface geometry from narrow to wide has a significant and reproducible effect on the value of the analysed parameter. The normality of differences between paired observations was assessed using the Shapiro–Wilk test, which indicated that the assumption of normality was not fully satisfied (W = 0.748, *p* = 0.0077). Despite this, a paired Student *t*-test was applied due to its robustness to moderate deviations from normality, particularly in cases with a large effect size and consistent differences between paired measurements. In the present study, the observed effect was substantial, which supports the validity of the parametric approach. Additionally, descriptive statistics, 95% confidence intervals, and effect size (Cohen’s dz) were reported to provide a comprehensive interpretation of the results.

The construction of the WPC profile means that screws are placed in areas where the profile has a chamber cross-section, and in such situations the screws are held only by the ‘ribs’ that thicken the profile precisely to increase its strength and enable the installation of fasteners in them. This means that the screws catch in the material only on these ‘ribs’ and not over the entire depth of the screwed part. This method of fastening screws makes it difficult to compare the results obtained with other materials that do not have a chambered structure and where the screws are held over the entire depth of the screw.

The topic of the screw-holding capacity of different materials has been appearing in scientific papers for over 50 years. To date, scientists have identified several factors that influence screw-holding capacity. These factors can be divided into two main groups. The first concerns the material and method of screw placement, and the second concerns the forces acting on these screws (static or dynamic loads and the direction of the force) [21,22]. The first, very broad group of factors includes, apart from issues related to the material in which the screws are placed (density, structure, IBS parameter, moisture content or age of the material) [22,23,24,25,26], those related to the screws themselves (dimensions, angles or other factors affecting the screw design) [17,27,28] and those related to the method of placing the screws in the material [29,30,31,32].

To compare materials’ screw retention capacity, the same screws and method of removal should be used. Among the studies conducted to date, it is difficult to find any that address the screw retention capacity of WPC profiles. There are no previous studies on SWR from WPC profiles. Scientists have determined the SWR of WPCs. Haftkhani et al. [33] studied the influence of screw type and size, pilot hole size, and screw insertion depth on WPCs and the results for WPC were compared with those for particleboard and MDF. The SWR for WPC was found to be higher than for these two types of boards. Ghanbari et al. [34] examined the influence of WPC composite manufacturing parameters on some of its mechanical and physical properties. However, these studies describe the composite as a solid material, so these results differ from those obtained for a profile made of WPC. In industrial practice, various profiles are used, and an additional difficulty is that each profile differs in both the cross-section and the exact composition of the material from which it is made. The most used type of screw for determining SWR is the so-called confirmat, a fastener often used in the furniture industry. The use of this fastener would allow a comparison of the screw-holding capacity of the tested WPC profiles with that of other materials used in the production of door frames, such as particleboard, MDF, or wood. However, the very structure and dimensions of this WPC profile used to produce door frames make it impossible to insert a confirmat screw into them for testing purposes.

The influence of individual factors on SWR has been the subject of numerous studies. In their study, Rajak and Eckelman [30] examined the effects of individual parameters on the pull-out force of screws in particleboard and MDF. They determined the impact of both screw dimensions and pilot hole dimensions on SWR. They found that the diameter of the pilot hole is closely related to the diameter of the screws. For screws with an external diameter of less than 6 mm, the pilot hole should have a diameter that is 70–75% of the screw diameter, and for larger screws with a diameter of more than 6 mm, the pilot hole should have a diameter that is 80–85% of the main thread diameter to ensure optimal screw pull-out force. Sydor et al. [35] studied the effect of the feed rate of pilot hole drilling on the ability to hold screws in particleboard. They found that as the feed rate during pilot hole drilling increases, the roughness of the pilot hole’s inner surface increases, negatively affecting the screw-holding capacity. In this study, for particleboard, which is also used in the production of door frames, values ranging from 1364 N to 1775 N were obtained, depending on the board type and the drill feed rate used to drill the pilot hole. In another study, Sydor et al. [36] determined the effect of drill bit quality on SWR for pine wood. These studies show that drill bit blunting negatively affects screw holding capacity. The study describes in detail how the sharpness of the pilot hole drill bit affects the quality of the pilot hole and, consequently, the screw-holding capacity. In these tests, the values obtained for pine wood were around 3000 N.

The above-described tests are only examples of the many factors that affect SWR. In the case of the material used in these studies, which has a chamber structure, the influence of, for example, the quality of the pilot hole will be less significant due to the WPC profile’s structure. WPC profiles were used in the production of door frames due to their water resistance; therefore, it is impossible to improve the screw-holding capacity in terms of material. To increase the pull-out strength of screws in this material, focus on the screw itself and its dimensions. This necessitates further research to determine what screw dimensions will provide the greatest strength necessary to pull out the screws fastening the fittings in door frames.

## 4. Conclusions

Test results show that the location of the screws in the WPC profile affects the SWR. The results obtained show that the structure of the WPC profile and the location of the screws affect the SWR. In the case of screws mounted in the beam, the SWR values are approximately half those obtained for the band fragments. This is influenced by the depth of screw insertion, which causes the screw to pass through a different number of ‘ribs’ of the chamber profile. Door frames made of WPC profiles are one of the elements of the door leaf–frame assembly that must meet specific requirements to be classified in the appropriate strength class. The only way to improve the strength of the door frame as a door assembly element is to improve its screw retention capacity, as this has been identified by the door manufacturer as the weakest point of this assembly. Further tests with different screws (different types, diameters or lengths) and selecting the size of the pilot hole should be carried out to determine the solution that ensures the highest SWR values. SWR results for WPC profiles are crucial for the functioning of the entire door frame. Without achieving appropriate value, the door frame will not be able to create a durable door system; the efficiency of their operation determines whether they obtain the appropriate mechanical class.

## Data Availability

The original contributions presented in the study are included in the article; further inquiries can be directed to the corresponding author.
